# Heparin‐Induced Thrombocytopenia and Thrombosis in Patients With or Without a Thrombophilia Background: A Systematic Review Involved 602 Cases

**DOI:** 10.1155/ijvm/9338124

**Published:** 2025-12-29

**Authors:** Guangyu Han, Siying Song, Shuling Wan, Mengqi Wang, Da Zhou, Xunming Ji, Ran Meng

**Affiliations:** ^1^ Department of Neurology, Xuanwu Hospital, Capital Medical University, Beijing, China, ccmu.edu.cn; ^2^ Division of Neurocritical Care and Emergency Neurology, Center for Genomic Medicine, Massachusetts General Hospital, Harvard Medical School, Boston, Massachusetts, USA, harvard.edu; ^3^ Advanced Center of Stroke, Beijing Institute for Brain Disorders, Beijing, China, bibd.ac.cn; ^4^ National Center for Neurological Disorders, Xuanwu Hospital, Capital Medical University, Beijing, China, ccmu.edu.cn

**Keywords:** arterial thrombosis, heparin-induced thrombocytopenia and thrombosis, primary thrombophilia, secondary thrombophilia, venous thrombosis

## Abstract

**Background:**

Heparin‐induced thrombocytopenia (HIT), often accompanied by thrombotic events and collectively referred to as heparin‐induced thrombocytopenia and thrombosis (HITT), is worthy of attention. Herein, we analyzed the features of HITT in patients with or without a thrombophilia background present prior to heparin exposure, aiming to identify and customize treatment.

**Methods:**

We searched PubMed and EMBASE to identify studies published up to November 2024, via the keywords “heparin,” “thrombocytopenia,” “thrombosis,” and “thrombophilia.” Case series and reports with confirmed HITT were included.

**Results:**

A total of 602 patients (277 males and 325 females with a mean age of 57.00 ± 17.20 years), reported in 481 papers, were carefully analyzed. The median time of thrombocytopenia and thrombosis onset postheparin exposure was Day 9 (5–12) and Day 9 (6–12), respectively. The abnormal platelet counts recovered by Day 5 (3–7) after the cessation of heparin. A more pronounced platelet count reduction was shown in patients with a thrombophilia background than those without this entity (*p* = 0.004). In this HITT cohort, patients with a thrombophilia background were more likely to develop newly formed venous thrombosis, whereas those without thrombophilia were more prone to arterial thrombosis after heparin use. Anti‐platelet factor 4 (PF4)/heparin antibody assay was the mainstream diagnostic method for HITT.

**Conclusions:**

HITT typically presents as newly formed venous thrombosis in patients with a thrombophilia background and as arterial thrombosis in those without thrombophilia. Whereby, screening for thrombophilia in patients with HITT is suggested, as well as considering prolonged alternative anticoagulation therapy in patients with a thrombophilia background.

## 1. Introduction

Heparin‐induced thrombocytopenia (HIT) is an immune‐mediated disorder caused by anti‐platelet factor 4 (PF4)/heparin antibodies. It is primarily characterized by a marked decrease in platelet counts following heparin exposure. In a subset of patients, HIT is accompanied by newly formed venous and/or arterial thrombosis, a condition referred to as heparin‐induced thrombocytopenia and thrombosis (HITT). HITT occurs in approximately 20%–64% of all HIT cases, and the detailed distinctions between them are summarized in Table S1 [1–3].

The recognition of HIT has evolved over the past five decades. The first cases of thrombocytopenia associated with heparin exposure were reported in the late 1950s and 1970s, when clinicians observed paradoxical thrombotic events during heparin therapy [4, 5]. In the early 1990s, the underlying immune mechanism was clarified with the identification of antibodies against PF4‐heparin complexes [6, 7]. These advances established HIT as a distinct prothrombotic immune disorder rather than a simple adverse drug reaction.

Heparin remains an essential drug in the implementation of some invasive examinations such as digital subtraction angiography (DSA) and the treatment of thromboembolic diseases. However, the occurrence of HIT or HITT underscores the need for careful recognition. The diagnosis of HIT/HITT requires the combination of clinical assessment and laboratory testing. The first warning signal is a remarkable decrease in platelet count (> 30%) or the appearance of newly thrombotic manifestations. When HIT/HITT is suspected, heparin should immediately be discontinued. Diagnosis is guided by clinical scoring systems such as the 4Ts scores, together with laboratory confirmation by immunoassays detecting anti‐PF4/heparin antibodies and functional platelet activation assays (e.g., heparin‐induced platelet aggregation [HIPA], serotonin release assay [SRA], or flow cytometry), when available [1–3]. Importantly, diagnostic identification can be retrospective, and if HIT/HITT is excluded, patients may safely benefit again from heparin therapy. However, in clinical settings, patients often experience thrombotic events prior to heparin use, especially during intravascular interventions following thrombotic episodes, whereby the symptoms of HITT are frequently unrecognized in time, resulting in poor clinical outcomes.

Thrombophilia, either primary or secondary, results from genetic variants of coagulation factors, inherited deficiencies of natural anticoagulants, or acquired clinical risk factors, leading to a predisposition to thromboembolic disease. Thrombophilia background may influence the course of HITT, including the severity of thrombosis and the overall clinical outcomes [8–11]. To systematically explore this influence, we reviewed papers published in the PubMed and EMBASE databases prior to November 2024 and analyzed the characteristics of HITT, including clinical manifestations, diagnosis, treatment, and outcomes. Furthermore, subgroup analysis was performed based on different thrombophilia backgrounds.

## 2. Methods

### 2.1. Search Strategy and Study Selection

This systematic review was registered in PROSPERO with the number CRD42023474089 and conducted in accordance with the Preferred Reporting Items for Systematic reviews and Meta‐Analyses (PRISMA) guidelines (Table S2). We searched the PubMed and EMBASE databases up to November 2024, which comprehensively encompass biomedical and clinical literature related to HITT with or without thrombophilia. Primary thrombophilia such as factor V Leiden (FVL) and prothrombin G20210A mutations, as well as inherited deficiencies of protein C, protein S, and antithrombin were confirmed by genetic testing. Secondary thrombophilia was recorded clinically according to medical history, including surgery, malignant tumor, pregnancy and perinatal period, and trauma. Medical subject headings and keywords were as follows: “heparin induced thrombocytopenia and thrombosis OR HITT OR heparin induced thrombocytopenia OR HIT” and “thrombophilia OR hypercoagulability OR hypercoagulable states”. The full search strategies are presented in Table S3.

Studies were eligible for inclusion if they met all of the following criteria: (1) case reports, case series, or retrospective studies with confirmed HITT and (2) each HITT case had complete information on demographic data, clinical manifestations, diagnosis, treatment, and outcomes. Studies reported without full texts or published in languages other than English were also eligible if an English abstract provided sufficient detail. Cases that were repeatedly reported or had incomplete information were excluded.

### 2.2. Data Extraction

Data from all enrolled studies were extracted by one author (G.H) and were crosschecked by another author (S.S). All studies were further summarized using the name of the first author, year of publication, study characteristics (sample size, study design, and country), demographic data (age and sex), and clinical characteristics (primary and/or secondary thrombophilia background, purposes, formulations and routes of initiated heparin, onset time of thrombocytopenia and thrombosis, percentage of platelet decrease, types and locations of HITT‐associated thrombosis, 4Ts scores, diagnostic tests, treatment of HITT, follow‐up duration, and clinical outcomes).

### 2.3. Statistical Analysis

All statistical analyses in this study were conducted by Social Science Statistical Software Package (SPSS) Version 28.0 program (IBM, United States). Continuous variables following normal distribution were calculated as mean ± standard deviation and compared by one‐way analysis of variance (ANOVA); otherwise, these data were presented as median (interquartile range) and assessed by nonparametric Kruskal–Wallis *H* test. Categorical variables were expressed as counts and percentages and Pearson′s chi‐square test or Fisher′s exact test was applied to evaluate differences. Two‐tailed *p* value < 0.05 was considered as statistical significance.

## 3. Results

### 3.1. Clinical Characteristics of HITT Patients

**Figure 1:**
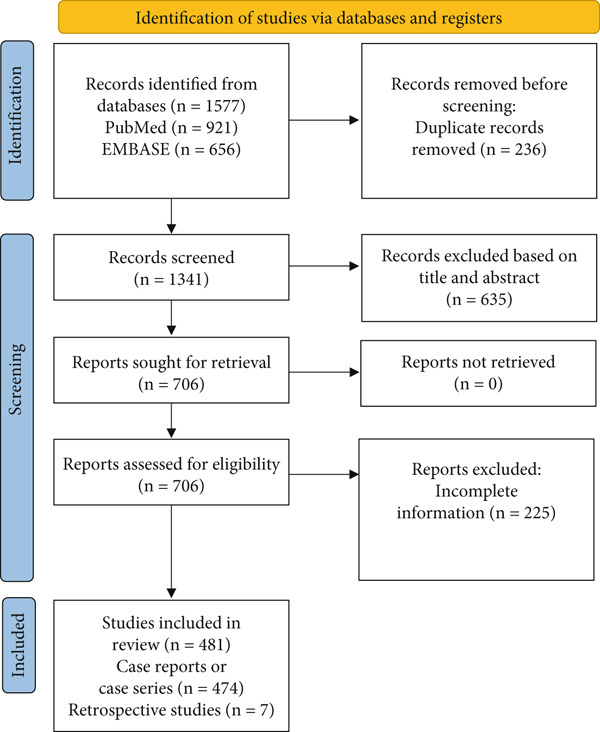
PRISMA flow diagram of the study selection process.

A total of 602 patients were finally selected from 481 published studies, including case reports and case series (*n* = 474) and retrospective studies (*n* = 7) (Figure 1). The included cohorts were sex‐balanced (277 males and 325 females) with a mean age of 57.00 ± 17.20 years. Cases from Western countries accounted for the majority (482/602, 80.1%). The top three comorbidities in HITT patients were obesity (148/602, 24.6%), high blood pressure (113/602, 18.8%), and Type 2 diabetes mellitus (80/602, 13.3%) (Table 1).

**Table 1:** Clinical characteristics of HITT patients with or without primary or secondary thrombophilia.

**Variables**	**Primary thrombophilia (** **N** = 24 **)**	**Secondary thrombophilia (** **N** = 401**)**	**Combined primary and secondary thrombophilia (** **N** = 17**)**	**Without thrombophilia (** **N** = 160**)**	**All (** **N** = 602**)**	**p**
Demographics						
Age(years) ^∗^	45.54 ± 19.34	59.18 ± 15.88^a^	37.65 ± 16.23	55.31 ± 18.13	57.00 ± 17.20	<0.001
Sex (male:female)	7:17	185:216	4:13	81:79	277:325	0.056^b^
Country^c^						
Western	17/24 (70.8%)	325/401 (81.0%)	11/17 (64.7%)	129/160 (80.6%)	482/602 (80.1%)	0.239
Eastern	7/24 (29.2%)	69/401 (17.2%)	6/17 (35.3%)	25/160 (15.6%)	107/602 (17.8%)	0.097
African	0/24 (0.0%)	7/401 (1.8%)	0/17 (0.0%)	6/160 (3.8%)	13/602 (2.1%)	0.462
Comorbidities^c^						
Obesity (BMI > 25 kg/m^2^)	5/24 (20.8%)	104/401 (25.9%)	3/17 (17.6%)	36/160 (22.5%)	148/602 (24.6%)	0.777
HBP	4/24 (16.7%)	75/401 (18.7%)	1/17 (5.9%)	33/160 (20.6%)	113/602 (18.8%)	0.579
T2DM	0/24 (0.0%)	58/401 (14.5%)	1/17 (5.9%)	21/160 (13.1%)	80/602 (13.3%)	0.163
Hyperlipidemia	3/24 (12.5%)	37/401 (9.2%)	0/17 (0.0%)	11/160 (6.9%)	51/602 (8.5%)	0.442
CHD	0/24 (0.0%)	28/401 (7.0%)	0/17 (0.0%)	5/160 (3.1%)	33/602 (5.5%)	0.197
COPD	0/24 (0.0%)	10/401 (2.5%)	1/17 (5.9%)	3/160 (1.9%)	14/602 (2.3%)	0.499

*Note:* Statistically significant values are given in bold.

Abbreviations: BMI, body mass index; CHD, coronary heart disease; COPD, chronic obstructive pulmonary disease; HBP, high blood pressure; HITT, heparin‐induced thrombocytopenia and thrombosis; and T2DM, Type 2 diabetes mellitus.

^∗^Mean ± standard deviation.

^a^Significant difference compared with other three groups.

^b^Pearson′s chi‐square test.

^c^Categorical variables are given as *n*/*N*, where *n* is the number of patients in which the variable was present and *N* the total number of patients for which that particular variable was reported.

With respect to the thrombophilia background, the majority of patients were with secondary thrombophilia (401/602, 66.6%) or without identifiable thrombophilia (160/602, 26.6%). Only a small proportion of patients had primary thrombophilia (24/602, 4.0%) or even primary and secondary thrombophilia (17/602, 2.8%). The most common types of primary thrombophilia were the FVL mutation (14/41, 34.1%), the prothrombin G20210A mutation (13/41, 31.7%), inherited protein S deficiency (10/41, 24.4%), and methylene tetrahydrofolate reductase (MTHFR) C677T mutation (6/41, 14.6%); whereas the major causes of secondary thrombophilia were surgery (334/418, 79.9%), malignant tumor (43/418, 10.3%), and pregnancy and perinatal period (17/418, 4.1%).

### 3.2. Heparin Use in Clinical Settings

#### 3.2.1. Purposes of Heparin Use

Heparin was administered either for prophylactic (329/602, 54.7%) or therapeutic purposes (273/602, 45.3%). The therapeutic use of heparin primarily targeted ischemic diseases, including venous thrombosis (145/273, 53.1%), cardiovascular disease (85/273, 31.1%), and arterial disease (43/273, 15.8%). Among patients with venous thromboembolism, the most common entities were deep venous thrombosis (DVT) (64/145, 44.1%), DVT with pulmonary thromboembolism (PTE) (37/145, 25.5%), and isolated PTE (25/145, 17.3%), whereas a few thrombi were located in the portal vein, cerebral venous system, or mesenteric veins. Cardiovascular and arterial indications included acute myocardial infarction (AMI), unstable angina pectoris, and thrombosis of limb or intracranial arteries (Table 2).

**Table 2:** The clinical spectrum of HITT patients with or without primary or secondary thrombophilia.

**Variables** ^**a**^	**Primary thrombophilia (** **N** = 24**)**	**Secondary thrombophilia (** **N** = 401**)**	**Combined primary and secondary thrombophilia (** **N** = 17**)**	**Without thrombophilia (** **N** = 160**)**	**All (** **N** = 602**)**	**p**
Purpose of initiated heparin						
Prophylactic	3/24 (12.5%)	260/401 (64.8%)^b^	6/17 (35.3%)	60/160 (37.5%)	329/602 (54.7%)	<0.001^c^
Therapeutic	21/24 (87.5%)	141/401 (35.2%)^b^	11/17 (64.7%)	100/160 (62.5%)	273/602 (45.3%)	<0.001^c^
Venous thrombosis	17/21 (81.0%)^d^	69/141 (48.9%)	11/11 (100.0%)^d^	48/100 (48.0%)	145/273 (53.1%)	<0.001^c^
DVT	7/17 (41.1%)	34/69 (49.3%)	3/11 (27.3%)	20/48 (41.6%)	64/145 (44.1%)	0.565
DVT + PTE	5/17 (29.5%)	18/69 (26.1%)	1/11 (9.1%)	13/48 (27.1%)	37/145 (25.5%)	0.652
PTE	1/17 (5.9%)	11/69 (15.9%)	1/11 (9.1%)	12/48 (25.0%)	25/145 (17.3%)	0.300
Portal vein thrombosis	3/17 (17.6%)^e^	2/69 (2.9%)	3/11 (27.3%)^d^	1/48 (2.1%)	9/145 (6.2%)	**0.003**
CVT/CVST	1/17 (5.9%)	3/69 (4.3%)	2/11 (18.1%)	1/48 (2.1%)	7/145 (4.8%)	0.178
Mesenteric venous thrombosis	0/17 (0.0%)	1/69 (1.5%)	1/11 (9.1%)	1/48 (2.1%)	3/145 (2.1%)	0.319
Cardiac vascular disease	1/21 (4.7%)	61/141 (43.3%)^f^	0/11 (0.0%)	23/100 (23.0%)	85/273 (31.1%)	<0.001
AMI	1/1 (100.0%)	38/61 (62.3%)	0/0 (0.0%)	15/23 (65.2%)	54/85 (63.5%)	1.000
UAP	0/1 (0.0%)	23/61 (37.7%)	0/0 (0.0%)	8/23 (34.8%)	31/85 (36.5%)	1.000
Arterial disease	3/21 (14.3%)	11/141 (7.8%)	0/11 (0.0%)	29/100 (29.0%)^e^	43/273 (15.8%)	<0.001
Limb arterial thrombosis	1/3 (33.3%)	6/11 (54.5%)	0/0 (0.0%)	26/29 (89.7%)	33/43 (76.7%)	**0.010**
TIA/stroke	2/3 (66.7%)	5/11 (45.5%)	0/0 (0.0%)	3/29 (10.3%)	10/43 (23.3%)	**0.010**
Formulations and routes of initiated heparin						
UFH	24/24 (100.0%)^g^	331/401 (82.5%)	12/17 (70.5%)	144/160 (90.0%)	511/602 (84.9%)	**0.004**
i.v.	23/24 (95.8%)	238/331 (71.9%)	11/12 (91.7%)	109/144 (75.7%)	381/511 (74.6%)	**0.021**
s.c.	1/24 (4.2%)	93/331 (28.1%)	1/12 (8.3%)	35/144 (24.3%)	130/511 (25.4%)	**0.021**
LMWH s.c.	0/24 (0.0%)^g^	70/401 (17.5%)	5/17 (29.5%)	16/160 (10.0%)	91/602 (15.1%)	**0.004**
Enoxaparin	0/0 (0.0%)	41/70 (58.6%)	3/5 (60.0%)	7/16 (43.8%)	51/91 (56.0%)	0.583
Dalteparin	0/0 (0.0%)	19/70 (27.1%)	2/5 (40.0%)	4/16 (25.0%)	25/91 (27.5%)	0.821
Nadroparin	0/0 (0.0%)	10/70 (14.3%)	0/5 (0.0%)	5/16 (31.2%)	15/91 (16.5%)	0.209
Thrombocytopenia onset time (days)^h^	8.50 (5.25–12.00)	9.00 (6.00–12.00)	8.00 (6.50–12.50)	9.00 (5.00–11.75)	9.00 (5.00–12.00)	0.848
HITT‐associated thrombosis onset time (days)^h^	8.50 (5.25–12.75)	9.00 (6.50–13.00)	8.00 (6.50–13.50)	9.00 (6.00–12.00)	9.00 (6.00–12.00)	0.742
Platelet counts decreased percentage (%) ^∗^	63.63 ± 17.73	68.24 ± 16.05	63.71 ± 16.71	62.79 ± 17.66^e^	66.48 ± 16.73	**0.004**
Platelet counts recovery time (days)^h^	4.00 (3.00–8.00)	5.00 (3.00–7.00)	4.00 (1.25–5.00)	5.00 (3.00–7.00)	5.00 (3.00–7.00)	0.089

*Note:* Statistically significant values are given in bold.

Abbreviations: AMI, acute myocardial infarction; CVST, cerebral venous sinus thrombosis; CVT, cerebral venous thrombosis; DVT, deep venous thrombosis; HITT, heparin‐induced thrombocytopenia and thrombosis; i.v., intravenous injection; LMWH, low molecular weight heparin; PTE, pulmonary thromboembolism; s.c., subcutaneous injection; TIA, transient ischemic attack; UAP, unstable angina pectoris; UFH, unfractionated heparin.

^∗^Mean ± standard deviation.

^a^Categorical variables are given as *n*/*N*, where *n* is the number of patients in which the variable was present and *N* the total number of patients for which that particular variable was reported.

^b^Significant difference compared with the primary thrombophilia group and the without thrombophilia group.

^c^Pearson′s chi‐square test.

^d^Significant difference compared with secondary thrombophilia group and without thrombophilia group.

^e^Significant difference compared with secondary thrombophilia group.

^f^Significant difference compared with other three groups.

^g^Significant difference compared with combined primary and secondary thrombophilia group.

^h^Median (interquartile range).

#### 3.2.2. Formulations and Administration Modes of Heparin

The majority of patients underwent unfractionated heparin (UFH) (511/602, 84.9%), especially during interventional procedures such as stent placement, balloon angioplasty, and embolectomy. The remaining patients were exposed to low molecular weight heparin (LMWH) (91/602, 15.1%), including enoxaparin (51/91, 56.0%), dalteparin (25/91, 27.5%), and nadroparin (15/91, 16.5%). UFH was predominantly administered via intravenous injection (381/511, 74.6%), whereas all LMWH doses were delivered subcutaneously (Table 2).

#### 3.2.3. Occurrence of HIT and HITT

The median time from initial heparin exposure to thrombocytopenia occurrence was 9 (5–12) days, and to thrombosis occurrence was also 9 (6–12) days. Subgroup analysis based on thrombophilia background revealed no significant difference. The platelet counts, as a vital and widely used marker for the diagnosis of HITT, decreased by 66.48 ± 16.73*%* overall compared with baseline. In subgroup analysis, the mean percentage of platelet decline was 63.63 ± 17.73*%* in patients with primary thrombophilia, 68.24 ± 16.05*%* in those with secondary thrombophilia, 63.71 ± 16.71*%* in those with both primary and secondary thrombophilia, and 62.79 ± 17.66*%* in those without thrombophilia. The decline was significantly greater in patients with secondary thrombophilia than in those without thrombophilia (*p* = 0.004). Heparin was promptly discontinued upon suspicion of HITT, and platelet counts gradually recovered to baseline within a median of 5 (3–7) days. Recovery duration was similar in patients with or without thrombophilia (Table 2).

#### 3.2.4. Locations and Types of Newly Formed Thrombosis in HITT

In most patients, the sites of HITT‐associated thrombosis differed from those of the original thrombotic events that occurred prior to heparin exposure (480/602, 79.7%), whereas in a minority of patients, both the new and original thrombi were located at the same sites (102/602, 17.0%) (Table 3).

**Table 3:** The locations and types of newly formed thrombosis of HITT.

**Variables** ^**a**^	**Primary thrombophilia (** **N** = 24**)**	**Secondary thrombophilia (** **N** = 401**)**	**Combined primary and secondary thrombophilia (** **N** = 17**)**	**Without thrombophilia (** **N** = 160**)**	**All (** **N** = 602**)**	**p**
HITT‐associated thrombosis locations						
Different locations from original thrombosis	9/24 (37.5%)^b^	347/401 (86.5%)	11/17 (64.7%)	113/160 (70.6%)^c^	480/602 (79.7%)	<0.001
Same locations as original thrombosis	11/24 (45.8%)	47/401 (11.7%)^d^	6/17 (35.3%)	38/160 (23.8%)	102/602 (17.0%)	<0.001
Both same and different locations from original thrombosis	4/24 (16.7%)^c^	7/401 (1.8%)	0/17 (0.0%)	9/160 (5.6%)	20/602 (3.3%)	**0.002**
HITT‐associated thrombosis types						
Isolated venous thrombosis	14/24 (58.4%)	213/401 (53.1%)	16/17 (94.1%)^b^	73/160 (45.6%)	316/602 (52.5%)	**0.002** ^e^
DVT	4/14 (28.6%)	94/213 (44.1%)	8/16 (50.0%)	50/73 (68.5%)^f^	156/316 (49.4%)	**0.001** ^e^
PTE	5/14 (35.7%)	76/213 (35.7%)	5/16 (31.2%)	13/73 (17.9%)^c^	99/316 (31.3%)	**0.031**
Visceral venous thrombosis^g^	2/14 (14.3%)	14/213 (6.6%)	2/16 (12.5%)	3/73 (4.1%)	21/316 (6.6%)	0.248
CVT/CVST	2/14 (14.3%)	16/213 (7.5%)	1/16 (6.3%)	2/73 (2.7%)	21/316 (6.6%)	0.230
Venous catheter thrombosis	1/14 (7.1%)	10/213 (4.7%)	0/16 (0.0%)	3/73 (4.1%)	14/316 (4.5%)	0.837
Venous graft thrombosis	0/14 (0.0%)	3/213 (1.4%)	0/16 (0.0%)	2/73 (2.7%)	5/316 (1.6%)	0.761
Isolated arterial thrombosis	5/24 (20.8%)	97/401 (24.2%)	1/17 (5.9%)	58/160 (36.3%)^c^	161/602 (26.8%)	**0.005**
Limb arterial thrombosis	3/5 (60.0%)	46/97 (47.4%)	1/1 (100.0%)	33/58 (56.9%)	83/161 (51.5%)	0.547
Stroke	1/5 (20.0%)	24/97 (24.7%)	0/1 (0.0%)	13/58 (22.4%)	38/161 (23.6%)	0.952
AMI	1/5 (20.0%)	18/97 (18.6%)	0/1 (0.0%)	3/58 (5.2%)	22/161 (13.7%)	0.067
Aortic thrombosis	0/5 (0.0%)	9/97 (9.3%)	0/1 (0.0%)	9/58 (15.5%)	18/161 (11.2%)	0.481
Combined venous and arterial thrombosis	5/24 (20.8%)	80/401 (20.0%)	0/17 (0.0%)	20/160 (12.5%)	105/602 (17.4%)	**0.030**
DVT + limb arterial thrombosis /stroke/AMI/aortic thrombosis	5/5 (100%)	52/80 (65.0%)	0/0 (0.0%)	11/20 (55.0%)	68/105 (64.8%)	0.177
PTE + limb arterial thrombosis /stroke/AMI/aortic thrombosis	0/5 (0.0%)	17/80 (21.3%)	0/0 (0.0%)	8/20 (40.0%)	25/105 (23.8%)	0.095
Venous graft thrombosis + limb arterial thrombosis/ stroke/AMI	0/5 (0.0%)	7/80 (8.7%)	0/0 (0.0%)	1/20 (5.0%)	8/105 (7.6%)	1.000
Visceral venous thrombosis + limb arterial thrombosis/ stroke/AMI	0/5 (0.0%)	4/80 (5.0%)	0/0 (0.0%)	0/20 (0.0%)	4/105 (3.8%)	0.656
Others^h^	0/24 (0.0%)	11/401 (2.7%)	0/17 (0.0%)	9/160 (5.6%)	20/602 (3.3%)	0.322

*Note:* Statistically significant values are given in bold.

Abbreviations: AMI, acute myocardial infarction; CVST, cerebral venous sinus thrombosis; CVT, cerebral venous thrombosis; DVT, deep venous thrombosis; HITT, heparin‐induced thrombocytopenia and thrombosis; and PTE, pulmonary thromboembolism.

^a^Categorical variables are given as *n*/*N*, where *n* is the number of patients in which the variable was present and *N* the total number of patients for which that particular variable was reported.

^b^Significant difference compared with the secondary thrombophilia group and the without thrombophilia group.

^c^Significant difference compared with secondary thrombophilia group.

^d^Significant difference compared with other three groups.

^e^Pearson′s chi‐square test.

^f^Significant difference compared with primary thrombophilia group and secondary thrombophilia group.

^g^Means adrenal, portal, splenic, and mesenteric veins thrombosis.

^h^Means atrial and/or ventricular thrombosis or cutaneous microvascular thrombosis leading to skin necrosis in the site of heparin injection.

The newly formed thrombosis presented as isolated venous thrombosis (316/602, 52.5%), isolated arterial thrombosis (161/602, 26.8%), and combined venous and arterial thrombosis (105/602, 17.4%). A small proportion of cases (20/602, 3.3%) manifested as atrial and/or ventricular thrombosis or cutaneous microvascular thrombosis leading to skin necrosis at the site of heparin injection. The common sites of isolated venous thrombosis were DVT (156/316, 49.4%) and PTE (99/316, 31.3%), whereas isolated arterial thrombosis primarily involved the limb, cerebral, cardiac, and aortic arteries. In more complicated cases where HITT‐associated thrombosis involved both veins and arteries, DVT combined with limb arterial thrombosis, stroke, AMI, or aortic thrombosis was commonly observed (68/105, 64.8%) (Table 3).

Subgroup analysis showed significant differences in the locations and types of HITT‐associated thrombosis. Patients with a secondary thrombophilia background were more likely to develop thrombosis at new sites distinct from their original ones (*p* < 0.001). Venous involvement was more frequent in patients with either primary or secondary thrombophilia (*p* = 0.002), whereas arterial thrombosis predominated among those without any thrombophilia background (*p* = 0.005) (Table 3).

### 3.3. Diagnosis of HITT

In clinical settings, a decreasing of platelet counts serves as the first warning signal of HITT; meanwhile, the development of newly thrombotic manifestations may also indicate disease onset. Confirmatory laboratory tests remain essential for diagnosis. In the present cohort, the diagnostic evaluation of HITT included immunoassays for detecting anti‐PF4/heparin antibodies and functional assays such as the HIPA and SRA tests. About half of the patients were diagnosed according to positive anti‐PF4/heparin antibodies (313/602, 52.0%), and 129 patients (21.4%) showed positive results in HIPA and/or SRA tests. A few patients (27/602, 4.5%) were diagnosed based on strong clinical suspicion, owing to negative or unavailable laboratory test results (Table 4).

**Table 4:** Diagnosis, treatment, and outcomes of HITT patients with or without primary or secondary thrombophilia.

**Variables** ^**a**^	**Primary thrombophilia (** **N** = 24**)**	**Secondary thrombophilia (** **N** = 401**)**	**Combined primary and secondary thrombophilia (** **N** = 17**)**	**Without thrombophilia (** **N** = 160**)**	**All (** **N** = 602**)**	**p**
Diagnostic tests						
Anti‐PF4/heparin Ab(+)	12/24 (50.0%)	213/401 (53.1%)	12/17 (70.5%)	76/160 (47.5%)	313/602 (52.0%)	0.274^b^
HIPA and/or SRA(+)	4/24 (16.7%)	83/401 (20.7%)	1/17 (5.9%)	41/160 (25.6%)	129/602 (21.4%)	0.228
Anti‐PF4/heparin Ab+HIPA+SRA(+)	6/24 (25.0%)	89/401 (22.2%)	2/17 (11.8%)	36/160 (22.5%)	133/602 (22.1%)	0.796
Anti‐PF4/heparin Ab+HIPA+SRA(−) or no tests	2/24 (8.3%)	16/401 (4.0%)	2/17 (11.8%)	7/160 (4.4%)	27/602 (4.5%)	0.217
Treatment						
Alternative anticoagulation therapy	24/24 (100.0%)	358/401 (89.3%)	16/17 (94.1%)	134/160 (83.8%)	532/602 (88.4%)	0.060
Direct thrombin inhibitors	15/24 (62.5%)	200/358 (55.9%)	6/16 (37.5%)	72/134 (53.7%)	293/532 (55.1%)	0.437^b^
Argatroban	7/15 (46.7%)	131/200 (65.5%)	4/6 (66.7%)	47/72 (65.3%)	189/293 (64.5%)	0.530
Lepirudin	7/15 (46.7%)	48/200 (24.0%)	2/6 (33.3%)	20/72 (27.8%)	77/293 (26.3%)	0.223
Bivalirudin	1/15 (6.6%)	21/200 (10.5%)	0/6 (0.0%)	5/72 (6.9%)	27/293 (9.2%)	0.870
Heparin derivatives or analogues	3/24 (12.5%)	79/358 (22.1%)	6/16 (37.5%)	31/134 (23.2%)	119/532 (22.4%)	0.328
Danaparoid	2/3 (66.7%)	46/79 (58.2%)	5/6 (83.3%)	21/31 (67.7%)	74/119 (62.2%)	0.604
Fondaparinux	1/3 (33.3%)	33/79 (41.8%)	1/6 (16.7%)	10/31 (32.3%)	45/119 (37.8%)	0.604
Oral anticoagulants	4/24 (16.7%)	57/358 (15.9%)	4/16 (25.0%)	22/134 (16.4%)	87/532 (16.3%)	0.753
Warfarin	4/4 (100.0%)	43/57 (75.4%)	3/4 (75.0%)	15/22 (68.2%)	65/87 (74.7%)	0.748
Rivaroxaban	0/4 (0.0%)	12/57 (21.1%)	0/4 (0.0%)	5/22 (22.7%)	17/87 (19.5%)	0.794
Dabigatran	0/4 (0.0%)	2/57 (3.5%)	1/4 (25.0%)	2/22 (9.1%)	5/87 (5.8%)	0.177
LMWH^c^	2/24 (8.3%)	22/358 (6.1%)	0/16 (0.0%)	9/134 (6.7%)	33/532 (6.2%)	0.786
Other adjunctive therapies^d^	0/24 (0.0%)	43/401 (10.7%)	1/17 (5.9%)	26/160 (16.2%)	70/602 (11.6%)	0.060
Complications	2/24 (8.3%)	34/401 (8.5%)	0/17 (0.0%)	10/160 (6.3%)	46/602 (7.6%)	0.629
Bleeding complications	2/2 (100.0%)	16/34 (47.1%)	0/0 (0.0%)	7/10 (70.0%)	25/46 (54.3%)	0.254
DIC	0/2 (0.0%)	18/34 (52.9%)	0/0 (0.0%)	3/10 (30.0%)	21/46 (45.7%)	0.254
Follow‐up time (months)^e^	5.00 (3.00–9.75)	7.00 (3.00–12.00)	6.00 (3.00–8.00)	6.00 (3.00–12.00)	6.00 (3.00–12.00)	0.658
Clinical outcomes						
No recurrent thrombocytopenia and thrombosis	16/24 (66.6%)	286/401 (71.3%)	16/17 (94.1%)	118/160 (73.7%)	436/602 (72.4%)	0.162
Amputation	6/24 (25.0%)	50/401 (12.5%)	0/17 (0.0%)	23/160 (14.4%)	79/602 (13.1%)	0.111
Mortality d/t HITT	1/24 (4.2%)	24/401 (6.0%)	0/17 (0.0%)	7/160 (4.4%)	32/602 (5.3%)	0.851
Mortality d/t other causes^f^	1/24 (4.2%)	41/401 (10.2%)	1/17 (5.9%)	12/160 (7.5%)	55/602 (9.2%)	0.702

Abbreviations: Ab, antibodies; d/t: due to; DIC, disseminated intravascular coagulation; HIPA, heparin‐induced platelet activation; HITT, heparin‐induced thrombocytopenia and thrombosis; LMWH, low molecular weight heparin; PF4, platelet factor 4; and SRA, serotonin release assay.

^a^Categorical variables are given as *n*/*N*, where *n* is the number of patients in which the variable was present and *N* the total number of patients for which that particular variable was reported.

^b^Pearson′s chi‐square test.

^c^Means dalteparin, enoxaparin, and nadroparin.

^d^Means thrombolytic agents (urokinase, streptokinase, ancrod, and recombinant tissue plasminogen activator), dextran, antiplatelet medications, and intravenous immunoglobulin.

^e^Median (interquartile range).

^f^Other causes include malignant tumor, multiple organ dysfunction syndrome, sepsis, and so on.

### 3.4. Treatment of HITT

The first step was to cease all forms of heparin exposure once HITT was suspected. The majority of patients subsequently underwent alternative anticoagulation therapy (532/602, 88.4%), whereas the remaining patients were treated with other adjunctive methods, including thrombolytic agents, dextran, antiplatelet drugs, and intravenous immunoglobulin (70/602, 11.6%). Direct thrombin inhibitors were the most frequently used anticoagulants (293/532, 55.1%), followed by heparin derivatives or analogues (119/532, 22.4%). In selected cases, oral anticoagulants or LMWH were also administered (Table 4).

### 3.5. Outcomes of HITT

A small proportion of patients (46/602, 7.6%) developed complications, including bleeding (25/46, 54.3%) and disseminated intravascular coagulation (21/46, 45.7%). All patients completed 6 (3–12) months of outpatient follow‐up after discharge. Favorable clinical outcomes were observed in the majority of patients (436/602, 72.4%). However, 79 patients (13.1%) required limb amputation due to new or progressive thrombosis, and 32 patients (5.3%) eventually died from HITT (Table 4).

## 4. Discussion

### 4.1. The Mechanisms of HIT and HITT as Well as Thrombophilia

**Figure 2:**
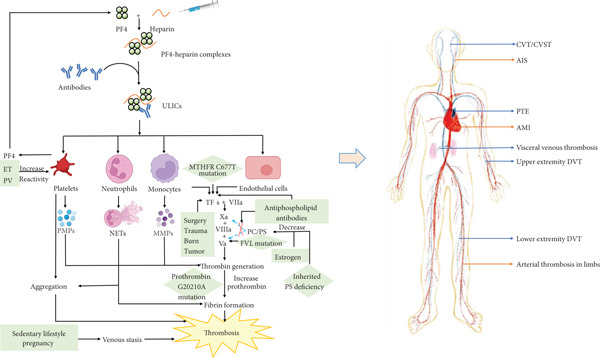
Mechanisms of HIT and HITT with thrombophilia background. HIT is an immune‐mediated condition caused by antibodies targeting PF4‐heparin complexes, which then form ULICs. Circulating ULICs promotes platelets and monocytes to release procoagulant PMPs and MMPs. Neutrophils are also activated by ULICs and then form NETs. Endothelial cells are injured after exposure to ULICs and subsequently produce TFs. Collectively, these responses trigger the coagulation cascade, resulting in thrombosis, namely HITT. Predisposing thrombophilia may further influence the development of HITT. Primary thrombophilia is presented in green rhombus boxes, and secondary thrombophilia is presented in green rectangular boxes. Blue dashed lines represent an inhibitory effect, and red “X” denotes inhibition. Abbreviations: AIS, acute ischemic stroke; AMI, acute myocardial infarction; CVST, cerebral venous sinus thrombosis; CVT, cerebral venous thrombosis; DVT, deep venous thrombosis; ET, essential thrombocythemia; FVL, factor V Leiden; HIT, heparin‐induced thrombocytopenia; HITT, heparin‐induced thrombocytopenia and thrombosis; MMPs, monocyte‐derived microparticles; MTHFR, methylene tetrahydrofolate reductase; NETs, neutrophil extracellular traps; PC, protein C; PF4, platelet factor 4; PMPs, platelet‐derived microparticles; PS, protein S; PTE, pulmonary thromboembolism; PV, polycythemia vera; TFs, tissue factors; ULICs, ultralarge immune complexes; Va, activated factor V; VIIa, activated factor VII; VIIIa, activated factor VIII; and Xa, activated factor X.

HIT is an immune‐mediated condition caused by heparin‐dependent antibodies that recognize PF4‐heparin complexes. The resulting ultralarge immune complexes (ULICs) bind to platelets via Fc gamma receptor IIA, triggering platelet activation and aggregation. This process leads to platelet consumption and a markedly increased risk of thrombosis. In addition, ULICs can promote monocytes, neutrophils, and endothelial cells to release procoagulant and prothrombotic microparticles, thereby further amplifying thrombus formation (Figure 2) [1–3, 12–24]. These prothrombotic mechanisms ultimately give rise to HITT.

In thrombophilia, prothrombotic responses may also be mediated through similar mechanisms. In secondary thrombophilia, surgical procedures can cause endothelial injury, and malignant cells in cancer patients induce procoagulant molecules expression. Myeloproliferative diseases such as polycythemia vera and essential thrombocythemia contribute to abnormal enhanced platelet reactivity [12, 15, 16]. In primary thrombophilia, inherited deficiencies of protein C, protein S, and antithrombin affect the hemostasis pathways related to factor V and prothrombin (Figure 2) [12, 25]. These primary and secondary thrombophilia may exert influence on the development of HITT through shared prothrombotic mechanisms. To our knowledge, this study is the first to integrate the clinical spectrum of HITT with different thrombophilia backgrounds.

### 4.2. Fluctuation of Platelet Counts in HITT Patients With Thrombophilia

Our study demonstrated that the median onset time of thrombocytopenia was at Day 9 (5–12) postheparin with a mean platelet reduction of 66.48 ± 16.73*%* among all HITT patients, and platelet counts returned to baseline levels within 5 (3–7) days after heparin cessation. These findings are consistent with previous reports, where thrombocytopenia typically occurred between Days 5 and 14 postheparin use, with platelet counts falling by 50% or more from baseline [13–19, 21, 26]. In most cases, platelet recovery to baseline required approximately 7 days after heparin was stopped [13, 23]. Whereby, dynamic monitoring of platelet counts prior to and postheparin is of great significance to recognize and evaluate the development tendency of HITT early.

Subgroup analysis revealed that HITT patients with a secondary thrombophilia background showed a more pronounced reduction in platelet counts compared with those without thrombophilia. This might be attributed to the increased risk of thrombosis in secondary thrombophilia, which enhanced platelet consumption. Although the timing of thrombocytopenia onset and platelet recovery following heparin cessation showed no significant difference, indicating that thrombophilia itself exerted limited influence on these parameters. Instead, anti‐PF4/heparin antibodies are likely to play a more critical role in the timeline of dynamic platelet counts.

### 4.3. Features of HITT in Patients With Thrombophilia

#### 4.3.1. Onset Time and Locations of HITT

During the process of HITT, thrombocytopenia typically occurs between Days 5 and 14 postheparin exposure, and thrombosis generally arises either simultaneously with thrombocytopenia or shortly thereafter [2, 3, 13, 21, 24, 27]. Our study demonstrated that the median onset times of thrombocytopenia and thrombosis were nearly identical following heparin use across all subgroups, and the thrombotic events rarely preceded the platelet decline, even in patients with primary and/or secondary thrombophilia backgrounds. Herein, we recommend that once patients manifest with decreased platelet counts postheparin use, prompt screening for thrombosis should be performed with the help of imaging, ultrasonography, or other methods. Considering HITT may develop at the site of original thrombosis prior to heparin use or at new sites, comprehensive screening at multiple locations is advocated for all patients with confirmed or strongly suspected HITT [14–16, 24, 28–30]. Especially for patients with secondary thrombophilia backgrounds, screening for thromboembolic events at new sites should be focused on.

#### 4.3.2. Types of HITT‐Associated Thrombosis

HITT can involve both the arterial and venous systems. In this study, we found that patients′ thrombophilia status prior to heparin exposure might affect the site of thrombosis when HITT developed [14, 15, 25, 31, 32]. HITT occurring in patients with either primary or secondary thrombophilia tended to present with venous thrombosis, whereas those without thrombophilia were more likely to develop arterial thrombosis. This difference may be associated with the distinct pathogenesis of venous and arterial thrombosis. Venous thrombosis is primarily driven by activation of the intrinsic or extrinsic coagulation pathways, and both primary thrombophilia (caused by genetic variants) and secondary thrombophilia (resulting from acquired risk factors) ultimately promote coagulation activation. In contrast, arterial thrombosis is typically initiated by rupture of an atherosclerotic plaque, which rapidly triggers platelet adhesion, activation, and aggregation at the ruptured site, leading to arterial occlusion [12, 15, 16, 25, 33].

### 4.4. Diagnosis and Exclusion of HITT

The diagnosis of HITT relies on a combination of clinical assessment and laboratory testing. The 4Ts scoring system is a simple and reliable tool for assessing the risk of HITT, which evaluates the magnitude, onset time, causative factors for thrombocytopenia, and thrombus formation postheparin use [1, 2, 12, 13, 18, 21, 23, 34, 35]. Patients with moderate and high risk scores require further confirmatory laboratory testing [23, 34, 35]. In patients with underlying thrombophilia, the diagnosis of HITT is particularly challenging because thrombotic events may occur independently of heparin exposure. In such cases, the characteristic pattern of platelet count decline following heparin administration, the temporal relationship between heparin exposure and thrombocytopenia, and the detection of anti‐PF4/heparin antibodies are crucial for distinguishing HITT from recurrent thrombotic events due to thrombophilia alone.

In this study, not all patients were evaluated according to the 4Ts scores, which were not specifically analyzed across different thrombophilia subgroups. The majority of HITT cases were diagnosed based on the presence of anti‐PF4/heparin antibodies, which provide high sensitivity but limited specificity. Other laboratory assays, such as the HIPA test and SRA, show high specificity but lower sensitivity [1, 2, 12–19, 21, 23, 34, 35]. Given the constraints of feasibility, sensitivity, and specificity among available assays, it is paramount to emphasize that the diagnosis of HITT still depends largely on strong clinical suspicion or a high 4Ts score, and a combination of multiple laboratory assays should be performed to improve the diagnostic accuracy [1, 14, 18, 34].

At the same time, it is equally important to recognize conditions in which HITT can be confidently excluded. A low 4Ts score (≤ 3) carries an excellent negative predictive value, making the diagnosis of HITT unlikely and thereby preventing unnecessary laboratory testing or inappropriate discontinuation of heparin [23, 34, 35]. In addition, the absence of new thrombotic events and negative anti‐PF4/heparin antibody results on enzyme‐linked immunosorbent assay (ELISA) can reliably support exclusion [35, 36]. More recently, a rapid and simple lateral flow immunoassay has been developed, which allows HITT to be ruled out within minutes [35, 37]. Incorporating these criteria into clinical practice helps to avoid overdiagnosis and inappropriate management.

### 4.5. Treatment and Duration of Anticoagulation in HITT Patients

All sources of heparin should be discontinued immediately in highly suspected HITT cases, and alternative anticoagulant or thrombolytic therapies should be initiated to prevent further thromboembolic events or to manage the established thrombosis [1–3, 12–21, 23, 34, 38, 39]. In this study, only 6.2% of patients received LMWH therapy, primarily documented in individual case reports or small case series, reflecting individualized clinical decision‐making or restricted drug availability [40–42]. Although LMWH is generally considered to carry a lower risk of inducing HITT than UFH, it can still trigger HITT or cross‐react with anti‐PF4/heparin antibodies, and therefore is not recommended as a first‐line treatment [3, 13, 17, 18, 43].

The majority of HITT cases in our cohort were treated with nonheparin anticoagulants. However, the duration of treatment was not consistently documented. Analyzing the available data, we found that the anticoagulation therapy was generally continued for at least 3 months in HITT patients. Based on different types of thrombophilia, patients with secondary thrombophilia are proposed to appropriately prolong the duration of anticoagulation until acquired risk factors are removed. For patients with primary thrombophilia caused by genetic variants, long‐term anticoagulation treatment beyond 6 months is generally recommended [23, 25]. In clinical settings, the duration of anticoagulant therapy should be tailored to individual patient characteristics.

## 5. Limitations

There were several limitations: Firstly, the results should be interpreted with caution, as most of our included studies were case reports or case series, which inherently introduce selection bias. Secondly, although some patients were reported without thrombophilia, the types of thrombophilia varied considerably, and certain forms—particularly primary thrombophilia—are difficult to identify, as their detection and confirmation depend heavily on the testing capacity of different laboratories. Consequently, the extracted information about thrombophilia might also exhibit biases. Moreover, some patients enrolled in our study had high 4Ts risk scores but without performed diagnostic tests, which might include certain misdiagnosed cases. Last but not least, we did not obtain complete 4Ts scores in all selected cases, resulting in incomplete data and limiting the feasibility of subgroup analyses.

## 6. Conclusions

In the development of HITT, thrombocytopenia and thrombus formation typically occur simultaneously. Dynamic monitoring of platelet counts postheparin use is essential for early recognition of HITT, alongside screening for thrombotic events with the help of imaging or other tools combined with clinical presentations. HITT usually presents as newly formed venous thrombosis in patients with a thrombophilia background, and arterial thrombosis in those without thrombophilia. Therefore, screening for thrombophilia in patients with HITT is recommended, and the duration of alternative anticoagulation therapy should be appropriately prolonged in patients with a thrombophilia background. In all HITT cases, anticoagulation therapy should be maintained for at least 3 months.

**Nomenclature**
AMI: acute myocardial infarction
DVT: deep venous thrombosis
FVL: factor V Leiden
HIPA: heparin‐induced platelet aggregation
HIT: heparin‐induced thrombocytopenia
HITT: heparin‐induced thrombocytopenia and thrombosis
LMWH: low molecular weight heparin
PF4: platelet factor 4
PTE: pulmonary thromboembolism
SRA: serotonin release assay
UFH: unfractionated heparin
ULICs: ultralarge immune complexes

## Disclosure

All authors read and approved the submitted version.

## Conflicts of Interest

The authors declare no conflicts of interest.

## Author Contributions

G.H. and S.S. wrote the first draft of the manuscript; G.H., S.S., S.W., M.W., and D.Z. performed material preparation, data extraction, and statistical analysis; R.M. wrote sections of the manuscript and contributed to manuscript revision; R.M. and X.J. contributed to the conception and design of the study; and R.M. took full responsibility for the data, the analyses and interpretation, and the overall conduct of the research. G.H. and S.S. contributed equally to this work.

## Funding

This work was funded by the National Natural Science Foundation of China (10.13039/501100001809, 82171297, 82101390); Central Guidance Fund for Local Science and Technology Development (ZYYD2025QY21); and Xuanwu Hospital Elite Cultivation Program (YC20250103).

## Supporting Information

Additional supporting information can be found online in the Supporting Information section.

## Supporting information


**Supporting Information 1** Table S1. Key differences between HIT and HITT.

**Supporting Information 2** Table S2. PRISMA 2020 checklist.

**Supporting Information 3** Table S3. Full search strategies.

## Data Availability

The datasets generated and/or analyzed during the current study will be made available from the corresponding author on reasonable request.
