# Supplementary material for: Heparin‐Induced Thrombocytopenia and Thrombosis in Patients With or Without a Thrombophilia Background: A Systematic Review Involved 602 Cases
Source: Int J Vasc Med. 2025 Dec 29;2025:9338124. doi: 10.1155/ijvm/9338124 (PMC12752825; doi:10.1155/ijvm/9338124)
Supplement: Supplementary file 1 — Supporting Information 1 Table S1. Key differences between HIT and HITT. [file IJVM-2025-9338124-s002.docx]

**Table S1. Key differences between HIT and HITT**

| **Items** | **HIT** | **HITT** |
| --- | --- | --- |
| **Definition** | An immune-mediated condition of heparin exposure characterized by platelet count reduction | HIT accompanied by new venous and (or) arterial thrombosis |
| **Incidence** | More common, overall incidence is 0.1-5% | Subset of HIT, accounting for about 20-64% of cases |
| **Clinical features** | Isolated thrombocytopenia after heparin exposure, typically >50% decline from baseline | Thrombocytopenia plus clinically significant thrombotic events |
| **Diagnosis** | Based on clinical assessment (e.g., 4Ts score), functional assays (HIPA and SRA), and immunoassays to detect anti-PF4/heparin antibodies | Same as HIT, but with objective evidence of new thrombus formation |
| **Prognosis** | Generally favorable with timely recognition and appropriate management | Higher mortality due to thrombotic complications, and requires urgent recognition and treatment |

**Abbreviations:** HIPA, heparin induced platelet aggregation; HIT, heparin induced thrombocytopenia; HITT, heparin induced thrombocytopenia and thrombosis; PF4, platelet factor 4; SRA, serotonin release assay.
