# Supplementary material for: Heparin‐Induced Thrombocytopenia and Thrombosis in Patients With or Without a Thrombophilia Background: A Systematic Review Involved 602 Cases
Source: Int J Vasc Med. 2025 Dec 29;2025:9338124. doi: 10.1155/ijvm/9338124 (PMC12752825; doi:10.1155/ijvm/9338124)
Supplement: Supplementary file 3 — Supporting Information 3 Table S3. Full search strategies. [file IJVM-2025-9338124-s001.docx]

**Table S3. Full search strategies**

Search included PubMed and EMBASE. Search date was from the inception through November 2024

1) PubMed search strategy

| 1. "thrombosis"[Mesh] |
| --- |
| 2. Venous thromboembolism [Title/Abstract] |
| 3. Venous thrombosis [Title/Abstract] |
| 4. Arterial thromboembolism [Title/Abstract] |
| 5. Arterial thrombosis [Title/Abstract] |
| 6. 1 OR 2 OR 3 OR 4 OR 5 |
| 7. "heparin"[MeSH Terms] |
| 8. "thrombocytopenia"[MeSH Terms] |
| 9. "heparin induced thrombocytopenia"[All Fields] |
| 10. "HIT" [All Fields] |
| 11. 7 OR 8 OR 9 OR 10 |
| 12. "thrombophilia" [Mesh] |
| 13. "hypercoagulability"[MeSH Terms] |
| 14. "hypercoagulable state"[All Fields] |
| 15. 12 OR 13 OR 14 |
| 16. 6 AND 11 AND 15 |

2) EMBASE search strategy

| 1. 'vein thrombosis'/exp |
| --- |
| 2. 'artery thrombosis'/exp |
| 3. Venous thromboembolisms:ab,ti |
| 4. Venous thrombosis:ab,ti |
| 5. Arterial thromboembolism:ab,ti |
| 6. Arterial thrombosis:ab,ti |
| 7. 1 OR 2 OR 3 OR 4 OR 5 OR 6 |
| 8. 'heparin induced thrombocytopenia'/exp |
| 9. heparin induced thrombocytopenia |
| 10. heparin induced thrombocytopenia:ab,ti |
| 11. heparin:ab,ti |
| 12. thrombocytopenia:ab,ti |
| 13. HIT:ab,ti |
| 14. 8 OR 9 OR 10 OR 11 OR 12 OR 13 |
| 15. 'thrombophilia'/exp |
| 16. 'hypercoagulability'/exp |
| 17. hypercoagulable state |
| 18. 15 OR 16 OR 17 |
| 19. 7 AND 14 AND 18 |
